# From Crescent to Mature Virion: Vaccinia Virus Assembly and Maturation

**DOI:** 10.3390/v6103787

**Published:** 2014-10-07

**Authors:** Liang Liu, Tamara Cooper, Paul M. Howley, John D. Hayball

**Affiliations:** 1 Experimental Therapeutics Laboratory, Hanson Institute and Sansom Institute, Adelaide, 5000, SA, Australia; E-Mails: Liang.Liu@unisa.edu.au (L.L.); Tamara.Cooper@unisa.edu.au (T.C.); Paul.Howley@sementis.com.au (P.M.H.); 2 School of Pharmacy and Medical Sciences, University of South Australia, Adelaide, 5000, SA, Australia; 3 Sementis, Ltd., Melbourne, 3000, Vic, Australia; 4 School of Medicine, University of Adelaide, Adelaide, 5005, SA, Australia

**Keywords:** vaccinia virus, virus assembly, membrane biogenesis, virion formation

## Abstract

Vaccinia virus (VACV) has achieved unprecedented success as a live viral vaccine for smallpox which mitigated eradication of the disease. Vaccinia virus has a complex virion morphology and recent advances have been made to answer some of the key outstanding questions, in particular, the origin and biogenesis of the virion membrane, the transformation from immature virion (IV) to mature virus (MV), and the role of several novel genes, which were previously uncharacterized, but have now been shown to be essential for VACV virion formation. This new knowledge will undoubtedly contribute to the rational design of safe, immunogenic vaccine candidates, or effective antivirals in the future. This review endeavors to provide an update on our current knowledge of the VACV maturation processes with a specific focus on the initiation of VACV replication through to the formation of mature virions.

## 1. Introduction

Vaccinia virus (VACV) is one of the most intensively studied members of the poxvirus family, primarily as a consequence of its unprecedented success as a live viral vaccine for smallpox, which mitigated eradication of the disease. Subsequent focus on VACV as a viral vaccine delivery platform has demonstrated great potential, and indeed, several experimental vaccines have been developed and tested in clinical trials including those for intractable infectious diseases, such as HIV and various therapeutic cancer vaccines [1,2]. Similar to other poxviruses, VACV is characterized by a large double-stranded DNA genome (192 kb), encoding for more than 200 proteins that function in virus entry, viral transcription, DNA and RNA synthesis, virion assembly and host immune suppression [3,4]. The large size of the VACV genome makes it highly amenable to transduction with multiple coding genetic inserts of almost limitless size and scope and because it resides in the cytoplasm there is little risk of inadvertent host genome integration events occurring. These fundamental and intrinsic characteristics of VACV, along with its proven capacity to generate lifelong and durable adaptive immune responses to encoded antigens, underpins its attractiveness as the basis of a robust vaccine vector platform technology.

Vaccinia virus has complex virion morphology. The replication cycle of VACV commences with activation of the viral gene expression programme, core dissolution and DNA replication before crescent membrane formation. Crescent membranes assemble into immature virions which then undergo virion maturation. These mature virions (MV) can then be wrapped to form enveloped MV (EV) [5,6]. The exact mechanisms of these processes are still not fully understood despite decades of intensive study. However, following recent technological advances, genetic, proteomic, molecular biologic and microscopic studies have provided a more detailed picture of VACV assembly. These studies have contributed to our understanding of the VACV maturation process which will ultimately facilitate the rational creation of safer and more efficient vaccine vector platforms and will also potentiate the discovery of new classes of anti-viral drugs [7].

In the last decade, two comprehensive reviews have detailed our current understanding of VACV structure and assembly [5,6], however, since then, advances have been made to answer some of the key outstanding questions, particularly the origin and biogenesis of the virion membrane, the transformation from immature virion (IV) to MV, and the role of several novel genes, which were previously uncharacterized, have now been shown to be essential for VACV virion formation. This review will endeavor to provide an update on our current knowledge of the VACV maturation processes with a specific focus on the initiation of VACV replication through to the formation of mature virions.

## 2. Entry of Vaccinia into Host Cells

Vaccinia virus has two main infectious forms, namely the MV and the extracellular EV. The virus can enter host cells by two routes, at a neutral pH viral membranes can directly fuse with cellular membranes while an acidic pH facilitates entry via the endosomal pathway [8,9]. Although largely unknown, different mechanisms are believed to be employed by MV and EV to enter the cell [10]. Endocytosis is triggered by virus-receptor interactions, followed by intracellular transportation before membrane fusion. Four viral proteins involved in VACV attachment to host cells have been identified, including A26, A27, H3, and D8, which bind with glycosaminoglycan or laminin on the cell surface [11,12,13,14,15]. Membrane fusion is believed to be mediated or catalyzed by a large macromolecular assembly of viral proteins in the MV, which has been named the entry fusion complex (EFC). This complex consists of nine transmembrane proteins: A16, A21, A28, G3, G9, H2, J5, L5 and O3 and two additional viral membrane proteins, F9 and L1, have been designated as EFC-associated [16,17,18,19,20,21,22,23,24,25,26,27,28,29]. Two recent reviews papers have detailed recent advances in our understanding of the cell entry process and have described the function of each of these proteins and associated complexes in detail [10,30].

## 3. Viral Factory Formation and Function

Following uptake of virus and dissolution of the core particle, “viral factories” are formed. These factories are concentrated infection-specific cytoplasmic domains of uniform density [31] and are believed to be the sites for viral assembly and viral DNA replication. The origin of the viral factory is still unclear. Immediately following cellular infection and before the appearance of any specific viral structures, these factories are transiently surrounded by cellular ER-derived membrane cisternae [31,32], which suggests the factories are initially defined by cellular components.

DNA replication and virion assembly occurs within viral factories [33,34] and co-located are the subsequently transcribed intermediate and late viral mRNAs. In addition, different lines of evidence, including the location of translation initiation factors and ribosomal proteins, and expression of different foreign proteins, support the notion that viral proteins are also translated within these factories [35]. Indeed, recent electron microscopy (EM) and tomography (ET) studies have definitively shown that the dense contents in these factories contain key proteins and membrane structures that are involved in membrane biogenesis, including pre-assembled D13 scaffolds, ruptured small ER membrane structures, and membrane assembly proteins [36,37].

## 4. Crescent Formation

The first distinguishable structures of virus morphogenesis are crescent membranes. In most electron micrographs, crescents are comprised of two distinct layers, an inner smooth layer that has the trilamellar appearance of a lipoprotein bilayer, and an external honeycomb lattice layer composed of trimers of the D13 protein [38,39,40,41]. Despite contradictory theories [38,40,42], most recent data from microscopic observations and biochemical, genetic, and topological studies, suggest the crescent membrane is clearly derived from the ER [36,43,44,45,46] rather than synthesized “*de novo*” [38]. The biogenesis process commences by rupture of the cellular membrane by VACV proteins functioning to generate an open membrane intermediate, which is stabilized by scaffold protein D13, and contributes to forming the crescent membrane [36,47]. At least nine genes have been shown to be essential for the formation of crescent membranes, including three structural components D13, A14, and A17, and six regulatory proteins, A6, A11, F10, H7, L2, and A30.5, which are all involved in membrane biogenesis and lately referred to as “viral-membrane assembly proteins”. The exact mechanism of how the viral membrane forms has not yet been resolved; however recent efforts are revealing how these proteins cooperate to complete the first stage of VACV assembly. 

### 4.1. Essential Structural Proteins for Crescent Formation: D13, A14 and A17

#### 4.1.1. The Scaffold of Crescent Membranes—D13

The scaffold protein D13 is expressed in the latter phase of infection. With a molecular weight of 62 kDa, it was the first gene identified as essential for membrane formation and it is now well characterized. In the 1960s, Dales and Mosbach described a “spicule” layer that comprised the convex surface of crescent and IV membranes [38]. Later on, three-dimensional deep-etch EM studies revealed the spicule layer as a continuous honeycomb lattice [48], consisting of D13 trimers [41] and arranged mostly in hexagons [49]. Several lines of evidence have demonstrated the essential role of D13 in VACV morphogenesis, including inhibition by the drug rifampin and inducible knockout mutant repression of D13 synthesis, both of which terminate virus replication [50,51,52]. In contrast, an amino acid change in D13 restores the functionality of D13 in the presence of the drug rifampin [53,54].

The D13 lattice acts as a mechanical scaffold for the growing crescent membrane, and its membrane re-modelling capacity facilitates maintenance of the constant radius of curvature until the membrane closes to form complete spheres [36,48,55]. The D13 lattice preassembles to form rod-like structures, and binds with the ER to form a “crescent precursor” [36,47]. One question that has been raised is that since D13 has no transmembrane domain, how can it associate with crescent membranes to form the lattice structure? Recent evidence has indicated that this could occur through interactions between D13 and the membrane protein A17, as both immunoaffinity purification and Western blot analysis demonstrates D13 can associate with A17 and immunogold electron microscopy also shows a close association of A17 and D13 in crescent membranes. Furthermore binding of A17 to D13 was abrogated by truncation of the N-terminal segment of A17 [56]. The D13 lattice remains associated with the developing virions until the complete assemble of IV, when it is removed to form the MV (discussed in a later section).

#### 4.1.2. Key Proteins Anchored in the Virion Membrane—A14 and A17

The structural transmembrane proteins A14, along with A17 are essential components of the virion membrane and are ~15 and 23 kDa respectively. They are both expressed late in infection and unlike D13 which is removed during the transformation of IV to MV, A14 and A17 are anchored and remain abundant in the MV membrane. Both *in vitro* and *in vivo* data has shown that A14 and A17 are synthesized in the membranes [43,57], and D13 interacts with A17 to form crescent precursors [58,59].

The A14 protein spans the viral membrane twice, having a topology where both the N- and C- termini face internally into the core, and the central hydrophilic loop is exposed to the outer surface [5,60]. It forms disulphide-linked homodimers via Cys^71^, and this dimerization enhances virion integrity and stability [61]. Previously, two A14 sites were thought to be important for its function in membrane biogenesis, including an N^83^HS motif that can be glycosylated and a Ser^85^ site that can undergo phosphorylation by F10 protein kinase, however, neither of these modifications has significant impact on A14’s biological competency [60]. Recently, two other crucial motifs have been identified that affect its biological function *in vivo*: N^9^YF which is located within the short hydrophilic region at the N terminus is important for maturation of IV to MV, whereas, P^39^TRTWK, within the hydrophilic loop that is exposed on the external surface of the virion membrane, is significant for both crescent enlargement and for MV production [59].

Similar to A14, A17 also spans the viral membrane twice, but with opposite polarities, where an internal hydrophilic loop extends into the luminal space and the N- and C- termini are exposed to the outer face of the virion membrane [57,62]. Both N- and C- termini undergo cleavage at an AG↓X motif, but at different stages of morphogenesis. The N-terminus regulates the association and disassociation of D13 protein, which are essential for crescent formation and subsequently the transition from IV to MV [56,59]. The C-terminus plays important roles in the maturation of IV to MV and in MV infectivity [56,59]. The F10-mediated phosphorylation on Ser, Thr, and Tyr residues within the C-terminal tail is vital for these bio-functions [63,64].

Repression of either A14 or A17 by inducible knockout leads to similar consequences, where crescent membrane formation is inhibited. Accumulations of large electron-dense virosomes, which contain viral membrane and core proteins, are observed in viral factories, and numerous clusters of 25 nm diameter membrane vesicles are found in the cytoplasm [59,61,65,66,67,68]. The exact composition of the vesicles is unclear, but evidence indicates they could consist of open membrane sheets which are formed by rupture of ER membranes [69]. Noticeably, the A17-deficent vesicles appear less dense and larger in size than those seen in A14-deficent infections [59], and this may be caused by differences in their function and/or structural-contributions to the membrane biogenesis.

### 4.2. Regulatory Proteins that are Essential for Viral Membrane Assembly

#### 4.2.1. Early Transcribed Membrane Regulatory Protein—L2

The L2 protein is 10.2 kDa and highly conserved in all chordopox viruses, but only very recently has been revealed as essential for formation of crescent membranes. Distinct from all other membrane-associated proteins, it is expressed early in VACV infection and remains stably-associated with the developing virion during the replication cycle, ultimately becoming a membrane component of the MV [70]. Suppression of L2 by a conditional lethal mutant exhibited defects in crescent membrane formation [70]. Using a recombinant VACV containing a tagged L2 protein, L2 was detected co-localized with the ER throughout the cytoplasm including in the viral factories, and was present at the edges of the crescent membranes [44]. More recently, a VACV L2R deletion mutant has been constructed and revealed that without L2, replication was aborted prior to mature virion formation [45]. Two types of aberrant structures were seen by EM, including short crescents located at the surface of viroplasm, which still contained proteins destined to form the cores of mature virions, and “empty” IV-like membranes, which appeared to be derived from smooth ER membranes. Together, this evidence suggests that L2 has an important role in recruiting the ER and modulating its transformation into viral membranes [45].

#### 4.2.2. A Small, L2-Interacting Protein—A30.5

The A30.5 protein was discovered only recently during purification of L2 proteins from VACV infected cells [46]. This ~5 kDa protein has a putative transmembrane domain, but no signal peptide or any conserved motif. Unlike L2, A30.5 is synthesized after DNA replication and co-localizes with the ER membrane and the L2 protein. An A30.5 deletion mutant has been constructed and in the absence of A30.5, VACV morphogenesis was interrupted. Large electron-dense cytoplasmic inclusions and clusters of D13-coated membranes that resembled crescents and immature virions devoid of viroplasm were observed. This study also provided further evidence supporting the ER origin of the VACV membrane, by showing that crescent-shaped membranes are contiguous with the ER membranes and oriented with the convex D13 lattice-coated side facing the lumen, while clusters of completed spherical IV-like structures are trapped within the expanded lumen. Therefore, the outer surface of the VACV virion is most likely derived from the luminal side of the ER membrane.

#### 4.2.3. A11, a Viral Factory-Located, and Membrane-Associated Protein

The 40 kDa A11 protein is expressed late in VACV infection and is localized in the viral factories but is not a significant component of the MV. The protein self-associates to form homodimers or higher order structures and is phosphorylated independently of the viral F10 kinase [71]. Repression of A11 by inducible knockout blocks VACV morphogenesis, resulting in the accumulation of large, dense bodies and a similar phenotype is observed as to when the other essential genes to crescent formation are interrupted, such as H7, L2 and A6 [71,72]. Recent studies show that A11 is localized with the ER within viral factories and present at the edges of crescent membranes in close association with A6 [72,73]. Similarly to L2, A11 is also involved in recruiting ER for virus assembly, but differently, A11 is expressed at late infection, and can only be found in the viral factory [72]. We speculate that A11 could be involved in stabilizing the crescent membrane ends to prevent resealing (fusion) before DNA uptake. This could explain the observation that large, dense bodies were accumulated after knockout of A11, as crescent precursors may reseal and accumulate to form the dense areas.

#### 4.2.4. Trafficking Protein A6

The 43 kDa A6 virion core protein plays an essential role in virion membrane biogenesis. It is expressed after viral DNA replication, and packaged in the core of the mature virion. Temperature–sensitive A6 mutants show that viral gene expression and viral factory formation is normal but virion morphogenesis is blocked [74]. A6 may also be involved in trafficking the membrane proteins to the virion assembly sites, as with repression of A6, some virion membrane proteins, such as A11, cannot localize in viral factories but are found scattered in the cytoplasm instead [73,75].

#### 4.2.5. H7, a Cytoplasmic Located Protein

Synthesis of the 17 kDa H7 protein occurs late during VACV infection, however, unlike other late expression proteins, H7 is not incorporated into mature virion [76]. This protein is also conserved in all vertebrate poxviruses, however it has no discernible functional motifs, nor non-poxvirus homologs. It localizes not only in viral factories but also throughout the cytoplasm, which suggests that H7 function is not limited to viral factories. Deletion of the H7 gene results in defective localization of D13 and the other MV membrane proteins to viral factories, however, the specific role of H7 is still unclear [37]. Notably, phenotypic differences were observed between the H7-null mutant and H7-inducible mutants, whereby crescent membranes were formed in iH7 mutant without inducing agent, but not in the ΔH7 infected cells, suggesting that leaky expression occurred from the un-induced mutant. This apparently aberrant result clearly demonstrates the crucial role that H7 plays in the development of short crescent membranes, and that even a small or undetectable amount of H7 could facilitate this process.

#### 4.2.6. Major Protein Kinase in the Virion Core—F10

The F10 protein is the major protein kinase found encapsulated in the virion core. It is a 50 kDa protein and expressed late during infection. Recombinant F10 protein has been shown to have kinase activity *in vivo* [77,78,79]. In addition, the F10 sequence has recognizable motifs associated with ATP binding and phosphotransfer, but other subdomains cannot be identified due to the high divergence from consensus motifs [80]. F10 is a dual-specificity protein kinase, which can directly phosphorylate serine, threonine, and tyrosine residues [64].

Expression of F10 protein is essential for VACV morphogenesis. Different F10-knockout mutant viruses have been constructed to characterize its biological function, including temperature-sensitive mutants and inducible knockouts [77,78,80,81]. Under non-permissive conditions, the F10 mutants show a profound defect in virion morphogenesis prior to crescent formation, implying that protein kinase activity is required for processing the essential proteins for membrane biogenesis. Indeed, as discussed earlier, A14 and A17, along with other possible membrane associated proteins, are phosphorylated by F10. Furthermore, F10 is not only involved in the early stages of virion assembly, but also plays important role in the transition of IV to MV (discussed later).

**Figure 1:**
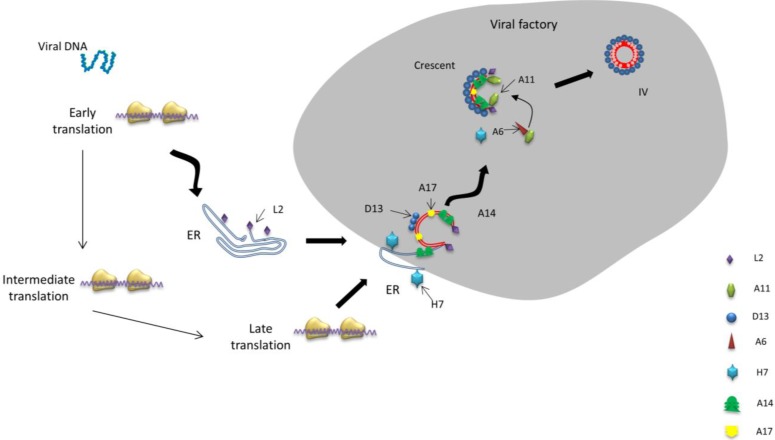
Model of Vaccinia virus (VACV) crescent membrane formation. Early transcribed protein L2 associates with the ER membrane and recruits it to viral factories, then L2 interacts with A30.5 or other unidentified proteins to rupture the ER membrane into small sections, while A14 and A17 are synthesized in the membrane in a co-translational fashion. Smooth membrane structures are recruited, opened up, and presented to pre-assembled scaffolds of D13, where D13 interacts with A17 to form crescent precursors. A11 stabilizes the end of the crescent to prevent it resealing, whereas A6 may play a role in transporting or controlling the transient association of A11 with the crescent end.

In summary, thus far, following viral fusion, the VACV genome is released and early transcription commences; L2 associates with the ER membrane and recruits it to viral factories, and possibly forms the boundary of the viral factory prior to producing crescent membrane; L2 interacts with A30.5 (or other unidentified proteins) to rupture the ER membrane into small sections which then form the smooth closed membrane structure. Prior to, or during, this process, A14 and A17 are synthesized in the ER. The smooth membrane structures are then recruited, opened up, and presented to pre-assembled scaffolds of D13, where D13 interacts with A17 to form crescent precursors. During assembly of the crescent membrane, A11 stabilizes the end of the crescent to prevent it resealing, whereas A6 may play a role in transporting or controlling the transient association of A11 with the crescent end, during crescent assembly and before sealing, to complete the spherical immature virion (Figure 1). However, further studies are required to verify this assumption and to shed light on the identity of possibly unknown genes that are involved in this process.

## 5. Immature Virion Formation

Crescents grow in length while maintaining the same curvature until they become closed circles or spheres in three dimensions, to form the immature virion (IV) [5,82]. Recent studies provide evidence that describes this process in further detail. Instead of growth in a continuous way, “independent crescents” or “crescent precursors” [36,47] assemble, possibly with the help of viral-membrane assembly proteins, into the spherical IV shape. As the crescent membranes develop, they are filled with viroplasm, which is known to contain viral core proteins and are uniform in density, but distinguishable from the surrounding factory [83]. Meanwhile, the viral genomic DNA is packed into the viroplasm before sealing of the IV membrane [36,82]. The detailed mechanisms of how the viroplasm associates with the growing crescent and how DNA is transported or incorporated into the virion remain unclear. However, a complex of seven VACV proteins has been identified that are required for this process to occur, along with regulatory protein E6 which is essential for core protein association.

### 5.1. Proteins Involved in Viroplasm-Membrane Association

#### 5.1.1. The Seven Protein Complex

A group of seven core proteins has been identified that form a multimeric complex and are essential for the association of viroplasm and viral membrane, [84,85] including A15, A30, G7, D2, D3, J1, and F10 [81,84,85,86,87]. The members of the complex interact and stabilize each other, and repression of any one these proteins results in destabilization of the others [81]. The structure of the complex is unknown, however, the polypeptides comprising the complex appeared to be in equimolar ratios, except for J1, which may be present as a homodimer [85,88].

The absence of any proteins in the complex exhibited similar phenotypes, in which apparently empty IVs with normal-looking viral membranes, accumulate and separate from the large masses of viroplasm [83,84,85,86,88,89]. These observations demonstrate their essential role in the process of viroplasm and membrane association; however, the detailed mechanism of how the complex functions is still unknown. Except for F10, the remaining six proteins do not have any recognizable structure/functional motifs which might imply that the complex plays a structural role. The obligatory role of F10 for A14 and A17 phosphorylation may indicate a potential interaction between the complex and the membrane protein. However, this requires further investigations

#### 5.1.2. E6 Is Also Involved in Membrane-Viroplasm Association

The E6 virion core protein is ~60 kDa and highly conserved in all chordopox viruses, but with no distinguishing functional motif and no homolog in non-poxvirus organisms [90]. Although the detailed function of E6 is still unclear, the current evidence shows that E6 acts on the same pathway as the seven-protein-complex, promoting the association of viroplasm with viral membrane crescents. E6 inducible KO mutants yield a phenotype virtually identical to the “seven protein complex” mutants, in which accumulation of both membrane crescent and viroplasm sub-assemblies are observed [90,91].

### 5.2. How the Genome Becomes Incorporated into the Virion Remains Unclear

Vaccinia genome DNA replicates in the cytoplasm and produces progeny genomes that will be incorporated into the virion [92]. The DNA is replicated as long concatemers that are resolved by the Holliday junction endonuclease A22 [93]. Genome replication is not coupled to VACV early morphogenesis, as genome maturation occurs normally even with interrupted crescent membrane formation [5]. The mechanism of how genome packaging into virions remains largely unknown. As discussed earlier, it is now clear that the genome DNA is trafficked into the IV before the sealing of the virion membrane [32,36,82]. However, several key questions are needed to be addressed in the future, what genes are involved in transporting the genome, what the mechanism for controlling genome copy number in the virion as the virus is large enough to accommodate multiple copies [94], and what is the signaling pathway for membrane sealing after genome encapsulation.

## 6. Transition of Immature Virus Particles to Mature Virions

**Figure 2:**
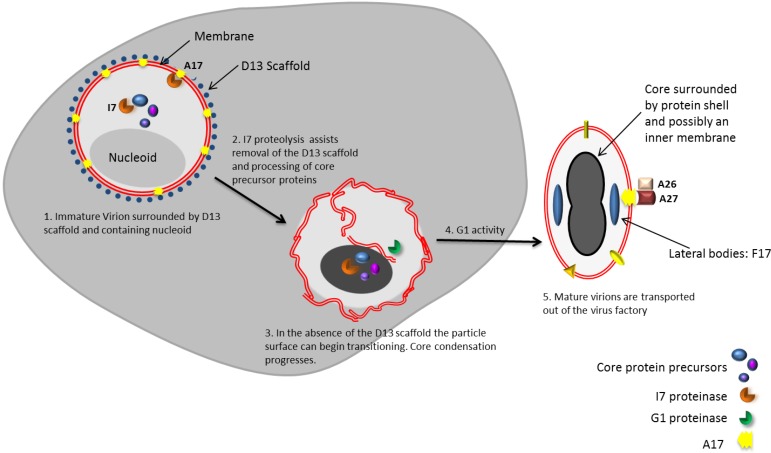
A summary of the transition of immature virion (IV) to mature virion (MV). I7 proteolysis has a role in removing the D13 scaffold by proteolytic processing of the membrane protein A17 (1). Core proteins are also cleaved by I7 into their mature forms (2). In the absence of the D13 scaffold the IV transitions towards becoming a MV and possibly involves rearrangement of the IV membrane into an inner and outer membrane (3). G1 activity is required to complete core condensation (4). The resulting MV contains a biconcave core that contains the viral genome and core proteins including the transcription apparatus required for early gene transcription. Lateral bodies are located outside of the core and enclosed by the MV membrane. The MV membrane contains additional proteins which can have functions that facilitate progression to enveloped MV (EV).

The next stage of morphogenesis transforms spherical IVs containing the nucleoid (IVNs) that are enclosed in a single membrane to form the first infectious particle, the intracellular MV. The MVs are characterized by a core that has biochemical and morphological features that are distinct from IVs. Two lateral bodies are observed either side of the core and these structures are enclosed by a lipid membrane to form the ‘brick-shaped’ MV. The proteome of MVs and IVNs are nearly identical with the exception of a few membrane proteins which are inserted during the transition of IVNs to MVs. This transition involves key proteolysis and redox reactions to remove the D13L scaffold and restructure the particle to a transcriptionally active and infectious virion (Figure 2).

### 6.1. Removal of the D13 Scaffold is Necessary but Not Sufficient for the Transition of IVs to MVs

The D13 scaffold that is characteristic of IVs no longer surrounds MVs. However, the signals for scaffold removal are not completely understood. Removal of the D13 scaffold correlates with proteolytic processing of A17 [56,59] and mutations that prevent cleavage of the N-terminus of A17 produce aberrant MV; they contain a core and appear ostensibly normal within what is described as a balloon-like circular membrane surrounded by D13 [59]. The processing of A17 has been shown to be dependent on I7, using temperature sensitive or inducible mutants [95]. The I7 protein is a core component that appears eight hours post-infection and shares homology with cysteine proteinases [96]. It is also responsible for processing the precursor forms of other core proteins (discussed later). In the absence of inducer, inducible I7 mutants fail to form MVs but result in the accumulation of IV, IVN and aberrant dense spherical particles that were identical in composition to MVs except for the absence of processed core proteins and that they remained associated with the D13 scaffold [95,96]. An inducible mutant of the A19 minor core component has shown that plaques and MVs do not form in the absence of inducer although the absence of A19 did not appear to affect the removal of D13 [97]. These cells contained only crescents, IVs and aberrant dense spherical particles that had little associated D13 scaffolding although they appeared to occasionally progress to being wrapped and exocytosed. Together, these examples serve to show that while removal the D13 scaffold is necessary, this alone is not sufficient for IVNs to progress to MVs.

### 6.2. Membrane Restructuring to Form MVs Remains Controversial

The MV membrane is distinguished from IVs by the presence of additional surface proteins. These proteins tend to have roles in cell attachment and mediating infection rather than morphogenesis but their presence can be considered important to the production of infectious MV and EV. Of recent note, while earlier roles of A17 have been discussed, its presence in the MV envelope later functions to anchor other viral proteins, such as A27 and A26, to the particle surface through direct or indirect interactions, respectively that is important for further development of MVs [98]. Membrane proteins A17 and A14 have been shown to associate with membranes *in vitro* in a co-translational manner only [59]. Their presence on both IV and MV membranes has suggested that the IV membrane remains at least part of the MV membrane. It is generally agreed that the intermediates between IVNs and MVs are short-lived as they are rarely observed and a number of models have been proposed to describe the processing of the IVN membrane to form MVs (reviewed in [5,6]). One debated feature of the models includes whether the core is surrounded by a second lipid layer and recent studies have furthered the argument for an inner membrane. Using electron tomography, distortions in the mostly continuous IV membrane have been observed [99]. At these points, the membrane fragments overlapped and were considered potential sites where crescents came together. Some IVNs showed areas of disordered D13 scaffold and in these regions, the membrane was described as having a waved aspect. A transient IVN-associated complex, speculated to have a role in maturation was also identified. The complex was usually located opposite the nucleoid attached to the inner side of the membrane with an extension that protruded outside of the virus particle. This study also identified putative intermediates that had a large number of discontinuities in the membrane and regions of overlapping membranes. Pieces of membranes also appeared to be forming core-like structures inside particles and areas of continuation between outer membrane pieces and internal membranes were observed. The absence of any evidence of continuity between the outer membrane and a lipid core wall had previously been used to favor the protein core wall model and the increased surface area of MVs compared to IVs was speculated to be accounted for by the insertion of membrane proteins or the presence of overlapping membranes that construct IVs. A recent study has imaged sub-strains of Western Reserve vaccinia with a number of accumulated mutations in the hope that there would be an increased amount of intermediate particles due to mutations blocking or decreasing the efficiency of virion morphogenesis. Similar fragmented IV membranes were detected and external pieces were seen connecting with internal structures that appeared to be developing a core shaped structure [32]. In summary, these studies propose that MVs contain external and internal membranes and that the inner membrane that surrounds the DNA core is the result of reorganization of the original IV membrane. It will be of interest to see these studies progress towards a working model explaining the signaling and protein interaction events that give rise to the intermediates described.

### 6.3. Transcriptional Activity May Be Linked to a Stable Core Structure

A protein shell surrounding the DNA has been evidenced by biochemical and imaging techniques but there is little understanding of the structure and organization within the core. Treatment with non-ionic detergents and reducing agents removes the outer membrane of virus particles to leave the cores intact. Prolonged protease treatment of these cores has produced AFM images of DNA spilling out of perforated or protease-damaged cores [100]. Inside of the proteinaceous shell, the genome is associated with DNA binding proteins along with the complete transcription machinery required for expression of early viral genes. If the transcription machinery is not packaged correctly and transcription is defective, the resulting MV may appear almost morphologically-normal but nevertheless are not infectious. Inducible mutant viruses deficient in core proteins L4/VP8 (DNA/RNA binding protein), L3 or H1 (protein phosphatase) have demonstrated this (reviewed in [5]) and likewise, temperature sensitive E8 mutants produced typical IV, IVN, and MV structures [101]. However, these virions were transcriptionally-defective so it is speculated that E8 may have a structural role that is directly or indirectly important for transcriptional activity. An essential subunit of the DNA-dependent VACV RNA polymerase is H4/rap94 and temperature sensitive or inducible H4 mutants failed to form plaques under non permissive conditions [102,103]. However, when infected cells were viewed by EM, what were thought to be morphologically-normal MVs were present, demonstrating that the core and lateral bodies can form independently of transcription machinery assembly. More recently, cells infected with inducible H4 mutant viruses were prepared using high pressure freezing, freeze substitution treatment and ultramicrotomy to produce TEM images that would allow a detailed visualization of the internal structure of virions [104]. Compared to wild-type infections, the absence of H4 produced virions that had an empty or collapsed core from which it was inferred that the transcription machinery is involved with forming a stable core structure.

### 6.4. Morphogenic Proteolysis Generates an Active Core

Similarly to other viral systems, proteolytic processing is essential in regulating morphogenesis, by providing a means of activating proteins at the appropriate time. This has been evidenced by the inhibition of proteolysis preventing infectious MV generation in the absence of changes to protein synthesis (reviewed in [105]). Sequence comparison and experimental data of precursor and processed core proteins A10, A3, and L4 identified a common AG↓X motif (where X can be A, S, or T, but not N) as the cleavage site and I7 was later identified as the core protease [106]. Although I7 is expressed late in infection, it has been identified in viral factories, IVs and MVs where it is confined to the core [107]. Proteolytic processing of major core proteins occurs only during the IV to MV transition but how I7 activity is regulated to achieve this is currently unknown. However, I7 alone is not responsible for core maturation. G1 is currently the only other VACV protein with similarity to other known proteases. It contains an HXXEH zinc-binding motif, which is an inversion of the motif characteristic of matrix metalloproteinases. The absence of G1 results in the arrested development of virus particles after core protein cleavage but before core condensation has been completed, resulting in a lack of MVs [108,109]. G1 itself is cleaved by a currently unidentified protease and the significance of this is also currently unclear [110]. Together the evidence from I7 and G1 mutants suggest that the proteases function sequentially since I7 mutants show arrest morphogenesis prior to core condensation, while the absence of G1 activity arrests morphogenesis after core condensation has commenced but prior to its completion.

### 6.5. Cellular Host Proteins Can Have Roles in Morphogenesis

Viruses are notorious for exploiting pre-existing host pathways for their own needs. Host protein, Golgin-97 is a component of MVs and has been shown to co-locate with insoluble core proteins while the N-terminus extends outside of the virus [111,112]. Using RNAi to suppress golgin-97 expression resulted in significantly decreased virus titers and inspection by EM showed the absence of MVs and the accumulation of IVs but further study is required to determine the how golgin-97 is involved in the transition of IVs to MVs [111].

Host cell phosphatidylinositol 3-kinases (PI3Ks) have also been shown to regulate the transition of IVs to MVs and also envelopment of extracellular MVs (discussed later). Two compounds; AS1 and AS2 from a kinase inhibitor library were found to significantly reduce plaque size [113]. The role of a host PI3K was then confirmed by infection studies using cell lines deficient in PI3K activity which reduced plaque number. The presence of AS1 reduced late protein production and trafficking of F13 which may contribute to the decreased number of MV identified by EM compared to in the absence of AS1.

Golgin-97 and PI3K are the most recent examples of the roles cellular proteins can have in virion morphogenesis. It will be of interest to observe what other host proteins will be found to be involved VACV morphogenesis in the future and how poxviruses exploit these host proteins to their own advantage.

### 6.6. The VACV Redox System is Important for Functional Membrane Proteins Displayed on the MV Surface

Repression of viral redox system proteins E10, A2.5 or G4 interrupts IVN transition to MV [114,115,116]. These proteins control disulphide bond formation in the typically reducing cytoplasmic environment and regulate the functions of a number of proteins including L1, A28, A21, L5 and H2. Common to these proteins is a transmembrane domain and localization to the MV membrane (reviewed in [117]).

### 6.7. Lateral Bodies Package Viral Proteins in Preparation for Delivery to the Cytosol of Host Cells

Complementing EM images, atomic force microscopy has also provided evidence for the presence of two lateral bodies (LBs) attached to opposing side of the MV core that were susceptible to protease treatment and may give the core its characteristic biconcave morphology [100]. There is little speculation on when and how these lateral bodies form and attach to the core. It has recently been proposed that lateral bodies facilitate the delivery of viral enzymes to the host cell cytosol [118]. This study used immunogold staining to identify F17 (also known as F18, p11, or VP11) as the main component of LBs although VH1 and G4 were also identified as being delivered into cells by LBs where proteasomes may contribute to LB disassembly upon which, F17 is rapidly degraded leading to speculation that F17 is a LB scaffold protein. These components are thought to account for only a fraction of the LB mass so other proteins that may modulate host cell processes or prevent virus recognition may become apparent in future studies. Curiously, repression of F17 from an inducible mutant has also been shown to generate only crescents and aberrant IVs with unusual internal membranes and nucleoids, morphologically-sound, infectious MVs were absent [119,120].

## 7. A Subset of MVs Go on to Form Enveloped Virions

Mature virions are transported out of viral factories and subsets of MVs are processed further to become enveloped virions (EVs) which support dissemination of the virus from cell to cell within an infected host. Microtubules traffic these MVs, which become wrapped in a double membrane derived from the trans-Golgi network or endosomes that incorporate viral proteins A33, A34, A36, A56, B5, E2, F12, F13, and K2. Wrapping and subsequent generation of EVs is known to require A27, B5 and F13 (also known as p37) (see [6] for review). A few studies have since expanded on the role of these proteins. F13 was recently shown to associate with late endosome host proteins and this interaction was required for plaque formation and wrapped virions [121]. A recombinant virus with B5 and A33 deleted, inhibited plaque formation almost completely whereas deletion of either B5 or A33 only reduced plaque size compared to the parent VACV [122]. The intracellular enveloped virus or wrapped virion can then fuse with the plasma membrane where it either remains associated with the cell or the formation of actin tails beneath the cell-associated EV promotes extracellular release of the virus or transmission to neighbor cells. Envelope proteins are thought to maintain the association of EVs with the cell and A36 was shown to promote release of EVs since certain amino acid substitutions abolished the commencement of actin tail formation and reduced EV release [123]. Cellular proteins FHOD1 and Rac1 have also recently implicated in actin tail formation and growth [124].

## 8. Conclusions and Open Questions

Clearly, the morphogenesis of VACV is a complex process. Different stages of morphogenesis can proceed concurrently and host proteins also have important roles in the process of forming crescents which assemble into IVNs and then progress to the infectious forms of VACV. Future advances in imaging techniques will likely resolve the sub-viral structure and further genetic and biochemical studies will increase our understanding of the protein interactions that progress and regulate morphogenesis. This intimate understanding of the morphogenesis of VACV will facilitate the future rational design of safe, immunogenic vaccine candidates or effective antivirals.
